# Biofeedback Training after Successful Inverted Internal Limiting Membrane (ILM)-Flap Technique for High Myopic Macular Hole

**DOI:** 10.3390/jcm12165188

**Published:** 2023-08-09

**Authors:** Alessandra Sborgia, Alfredo Niro, Valentina Pastore, Valeria Albano, Giacomo Boscia, Marina Piepoli, Camilla Di Pardo, Lorenzo Accurso Tagano, Marta Zerbinati, Luca Landini, Maria Grazia Pignataro, Giovanni Petruzzella, Rossella Donghia, Abdullah S. Alqahtani, Marco Coassin, Roberto Dell’Omo, Francesco Boscia, Giovanni Alessio, Giancarlo Sborgia

**Affiliations:** 1 Eye Clinic, Department of Medical Science, Neuroscience and Sense Organs, University of Bari, 70124 Bari, Italy; 2 Eye Clinic, “SS. Annunziata” Hospital, ASL Taranto, 74100 Taranto, Italy; 3 National Institute of Gastroenterology “S. de Bellis” Research Hospital, 70013 Castellana Grotte, Italy; 4 Department of Surgery, Division of Ophthalmology, National Guard Hospital, Jeddah 31982, Saudi Arabia; 5 King Saud bin Abdulaziz University for Health Sciences, Jeddah 22384, Saudi Arabia; 6 King Abdullah International Medical Research Center, Jeddah 22384, Saudi Arabia; 7 Ophthalmology, University Campus Bio-Medico, 00128 Rome, Italy; 8 Department of Medicine and Health Sciences “Vincenzo Tiberio”, University of Molise, 86100 Campobasso, Italy

**Keywords:** high myopic macular hole, inverted flap technique, microperimeter, biofeedback, retinal sensitivity, fixation

## Abstract

Background: Microperimetric biofeedback training improved visual acuity and fixation stability in patients who previously underwent macular surgery. We aimed to compare the functional results of biofeedback training with the standard of care in patients who underwent successful inverted Internal Limiting Membrane (ILM)-flap technique for high myopic macular holes (hMMH). Methods: This was a retrospective, comparative, cohort study. Patients with hMMH after surgical hole closure underwent microperimetric biofeedback using structured light stimulus plus acoustic tone (n = 12; Biofeedback) or standard of care with scheduled visits (n = 11; Control). Best-corrected visual acuity, retinal sensitivity at central 12° (RS) and 4° (CRS) with a mean deviation at central 12° (MD), and fixation stability as bivariate contour ellipse area (BCEA 68%, 95%, and 99%) were assessed at baseline and month 1, 3, 6, and 12. The Mann–Whitney test was used to test the difference between the groups. Results: Baseline functional parameters were not significantly different among the groups. BCVA significantly improved in each group (Biofeedback, *p* = 0.002; Control, *p* ≤ 0.02) at all follow-up visits. CRS significantly improved at 6 (*p* = 0.03) and 12 (*p* = 0.01) months in the Biofeedback group and at month 12 (*p* = 0.01) in the Control group. RS (*p* = 0.001) and MD (*p* = 0.005) improved at the last follow-up only in the trained group. After training, BCEA 68% and 95% significantly improved (6 and 12 months, *p* < 0.05). The Biofeedback group had better results in RS (*p* ≤ 0.02), CRS (*p* ≤ 0.02), and BCEA 68%, 95%, and 99% (*p* ≤ 0.01) compared to the Control at all follow-ups. BCVA and MD were better in the Biofeedback group at month 3 (*p* = 0.01), and month 3 (*p* = 0.01) and 12 (*p* = 0.003), respectively. Conclusions: Microperimetric biofeedback can increase retinal sensitivity and stabilize fixation better than the standard care over months after a successful inverted ILM-flap for hMMH.

## 1. Introduction

Macular holes (MHs) are a known clinical finding in patients with high myopia [1], with a prevalence of 8.5% [2], and the age at the onset of high myopic MH (hMMH) significantly decreases with the increase in myopic refraction [1]. Surgical intervention for hMMH is recommended when macular traction and visual acuity impairment progress [3]. The inverted internal limiting membrane (ILM)-flap surgical approach has demonstrated its effectiveness in terms of anatomical closure rate and visual acuity recovery compared to other techniques, including the traditional ILM peeling and the autologous transplantation of ILM [4,5,6]. The ILM-flap technique provides a high closure rate, ranging from 91.8% to 97.1%, but the pooled visual acuity improvement rate only ranges from 66.2% to 77.3% [7]. In addition, patients often naturally discover ways to adapt their visual system, such as shifting their focus to a non-central point (known as the Preferred Retinal Locus or PRL) to work around a macular hole scotoma [8,9,10,11]. However, the PRL location is not always ideal for optimal visual performance [12], as it may be in areas with low retinal sensitivity far from the center of the eye [13]. Additionally, fixation stability is weaker when focusing on peripheral areas compared to the center [14,15], and unstable fixation has been linked to reduced visual acuity [9,16,17]. Microperimetry was used to assess quantitative measures of macular function including retinal sensitivity and fixation behavior that may precisely correlate macular morphology and related function when assessing the outcomes of high myopic macular hole treatments [18]. The technique of visual rehabilitation has been extensively used to treat eye conditions that involve visual decline and instability in central fixation [19]. Biofeedback training improved eccentric vision, reinforcing a trained retinal locus (TRL) closest to the fovea and with the highest retinal sensitivity, that was selected from among the PRL spontaneously created and used for fixation by the patient [20]. Previous studies have shown that using biofeedback techniques that combine acoustic tones and structured light stimuli can improve visual function, reading speed, fixation behavior, and retinal sensitivity [21,22,23,24]. This technique may also improve communication between intraretinal neurons and support a “remapping phenomenon” in the retina–brain connection [19,25,26,27]. In patients who previously underwent macular surgery, microperimetric biofeedback training improved fixation stability and visual acuity [20] up to 6 months [20,28,29]. In this study, we compared the functional results of patients receiving biofeedback rehabilitation with those of patients receiving standard of care after successful vitrectomy and inverted ILM-flap technique to repair high myopic macular holes.

## 2. Materials and Methods

### 2.1. Study Design and Objectives

We conducted a single-center, retrospective, comparative cohort study on 23 patients affected by hMMH who achieved successful hole closure with the inverted ILM-flap technique. Before undergoing surgical treatment, all participants were required to read and sign a written informed consent form. In all cases, surgery was performed at the Eye Clinic of the University of Bari, Bari, Italy, between April 2019 and April 2022. All the surgeries were performed by the same experienced retinal specialist (GS). The surgical success was identified by the feature of the complete disappearance of a closed full-thickness macular hole and absence of neurosensory defect over the fovea, revealed by optical coherence tomography (OCT) scans [30]. After the operation, 12 patients underwent a standardized biofeedback rehabilitation protocol (Biofeedback) using MP-1 Microperimeter (MP-1, Nidek Technologies, Padova, Italy) while 11 were followed up with standard care (Control). All patients in the Biofeedback group gave informed consent for the rehabilitation protocol. The inclusion criteria were 18 years old or older; a high myopia, defined as an axial length greater than 26.5 mm [axial length was measured with a Zeiss IOLMaster 500^®^ (SNR > 200)] and/or refraction (spherical equivalent) over −6.00 diopters; successful closure of hMMH achieved with a single operation confirmed with OCT at 1 month after surgery; Best-corrected visual acuity (BCVA) better or equal to 1 logMAR after surgery; and a follow-up period ≥ 6 months. The exclusion criteria were amblyopia, corneal disease, a subcortical cataract or cataract with more than 3 nuclear scleroses or cortical opacity [31], glaucoma or ocular hypertension, diabetic retinopathy, retinal vascular disease, age-related macular degeneration, choroidal neovascularization, pre-surgical traumatic macular hole, pre-surgical macular hole complicated by foveoschisis or retinal detachment, a minimum diameter of the hole > 1000 μm, and signs of severe chorioretinal atrophy involving the fovea like the absence of outer retinal layers and backscattering around the macular hole evaluated by spectral-domain OCT (SD-OCT). The study protocol was approved by the ethics committee (IRB) of the Eye Clinic, University of Bari, Bari, Italy (according to the Declaration of Helsinki and its later amendments) in 2021 (Project identification code: 01-12/21). According to the Italian law for retrospective studies, the patients’ non-opposition is sufficient to process retrospective data.

### 2.2. Assessments

All patients were assessed at 1 month post-surgery, as the baseline time point, and at months 3, 6, and 12. During the visit, BCVA was measured with a standardized Early Treatment Diabetic Retinopathy Study (ETDRS) protocol; ETDRS values were converted to the logarithm of the minimum angle of resolution (logMAR) for statistical analysis; retinal sensitivity and fixation behavior were measured by an MP-1 microperimeter (MP-1, Nidek Technologies, Padova, Italy). Sensitivity was measured using a stimulus of 0.4 degrees, presented for 200 ms. Mean retinal sensitivity (RS), the mean sensitivity of all 45 loci in the central 12°, and mean central retinal sensitivity (CRS), and the mean sensitivity of the central 13 loci within central 4° were recorded. The threshold at each point was determined using a 4-2 staircase. The mean deviation (MD) was calculated by the MP-1 microperimeter software after a comparison of the measured retinal sensitivity with a normative database [32]. The “follow-up” feature of the software was used to obtain measurements at the same retinal sites during all visits. Fixation stability was recorded during the light sensitivity examination [33]. The bivariate contour ellipse area (BCEA) parameter was applied to enable collection of quantitative data on fixation stability in three concentric ellipsoid areas containing 68%, 95%, and 99% of the fixation points. To measure eye fixation, BCEA uses Cartesian axes to plot the position of each fixation and calculates the area of an ellipse that covers a set percentage of fixation points. This calculation relies on the standard deviations of horizontal and vertical eye movements during fixation [34,35]. A red cross with a 1° arm extension was used as the fixation target, but it was increased to ≥2° if patients could not see it [29,33,36]. Before beginning the examination, a 2 min demonstration pre-test was performed to avoid a learning effect. An auto-tracking system was used to calculate the horizontal and vertical shifts from the reference recording of the fixation area. Examinations that took longer than 15 min were excluded from the study.

### 2.3. Surgical Technique

During the surgeries, a retrobulbar block was administered using a mixture of 2% Lidocaina and 2% Mepivacaina, and the Constellation vitrectomy system from Alcon in Fort Worth, TX, USA was used. Phacoemulsification was performed on all phakic eyes, and all patients underwent a 27-gauge transconjunctival sutureless vitrectomy with a posterior vitreous detachment using either a soft silicone-tipped cannula or active suction with the vitrectomy probe. The macula area was stained with Brilliant Blue G to facilitate ILM peeling using an inverted ILM-flap technique, which was based on the original description by Michalewska et al. [37] with some modifications. To treat the macular hole, we used the pinch and grasp technique to remove the ILM up to about 2 disc diameters around it. We trimmed the edges of the ILM with a cutter and then flipped it over to cover the hole. During the air–fluid exchange and flap inversion, we reduced the perfusion pressure. The medical procedure involved the use of 22% SF6 gas tamponade, and patients were advised to maintain a face-down position for three days after the operation.

### 2.4. Biofeedback Strategy

In the Biofeedback group, after recording baseline BCVA, RS, CRS, MD, and BCEA at 1 month after surgery, all the patients underwent the same biofeedback rehabilitation protocol using an MP-1 Microperimeter [29] after signing the informed consent form. The protocol consisted of 12 training sessions, each lasting 10 min, twice a week. All functional tests were performed with the patient’s best correctable prescription employed. The fellow eye was occluded using a simple eye patch. During each session, patients were asked to move the eye according to audio feedback and a standardized, structured, and flickering light stimulus (a checkboard pattern with low spatial black/white elements with a size of about 0.5° on the fixation target), which advised patients when they were getting closer to the selected site of fixation. The PRL was considered more suitable for training than the aforementioned TRL, which was chosen by the ophthalmologist, preferring the PRL naturally developed by the patient within an area of fair retinal sensitivity. The frequency of the auditory signal increased and became continuous with the approach of fixation toward the TRL. Simultaneously, the flickering structured pattern was projected on the TRL instead of the fixation target. The fixation on the TRL had to be maintained as long as possible. The best TRL to be trained should have been located as close as possible to the scotoma, on the superior retinal field, with appropriate retinal sensitivity to ensure the reinforcement of fixation, as demonstrated by Nilsson [38].

### 2.5. Statistical Analysis

Statistical analysis was based on all patients included in the study. Mean and standard deviation for continuous variables, and relative frequency for categorical were used as indices of centrality and dispersion of the variable distribution. The non-parametric Chi-square test and Wilcoxon rank-sum test (Mann–Whitney test) were used to test the difference between the two groups. A Wilcoxon matched-pairs signed-rank test was performed on the changes in functional parameters of each group over follow-up. All statistical tests were performed at the *p* < 0.05 significance level. All the statistical computations were performed using StataCorp, 2015, Stata Statistical Software: Release 14. College Station, TX, USA: StataCorp LP.

## 3. Results

The demographic and pre-surgical data are reported in Table 1.

**Figure 1:**
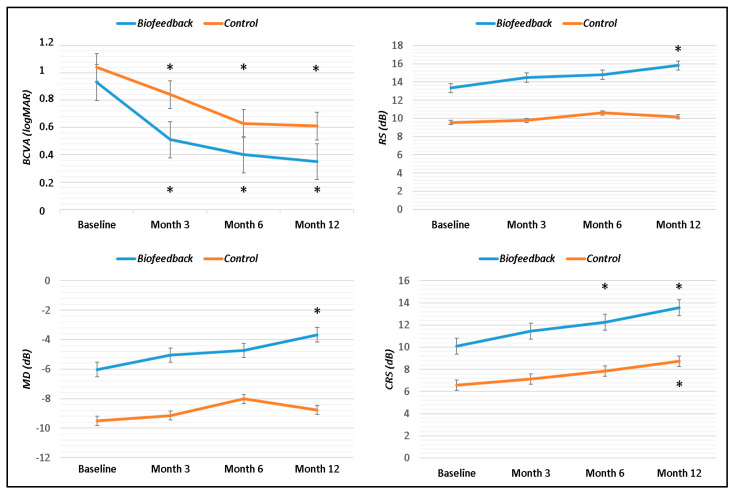
Changes in visual acuity and retinal sensitivity in Biofeedback and Control group. In both groups, a significant improvement in visual acuity (BCVA) was observed at all time points. In the Biofeedback group, all parameters of retinal sensitivity (RS, MD, CRS) significantly improved after training. In the Control group, only the sensitivity at central 4° (CRS) improved. Each point with a vertical bar represents the mean ± standard deviation. (BCVA, Best-corrected visual acuity; RS, retinal sensitivity; MD, mean deviation; CRS, central retinal sensitivity); * *p* < 0.05 within each group.

The study enrolled 23 patients, 12 who underwent biofeedback rehabilitation training and 11 who were followed with standard care. Overall, there were 14 males and 6 females. The overall mean age was 65.5 ± 8.2 years. The age of the patients ranged from 51 to 79 years in the Biofeedback group and 57 to 76 years in the Control group. There was a statistical difference in gender and axial length between the groups (*p* = 0.04) but not for age, hole size, lens status, and refractive error of phakic eyes. Furthermore, there was no statistical difference in pre-surgical BCVA between the two groups. During the 12-month follow-up, there were no dropouts in both groups. Over follow-up, in all cases, the hole closure was confirmed by OCT scans. BCVA not significantly improved after surgery when we compared the pre-surgical to baseline visual acuity in each group (Biofeedback: 1.03 ± 0.11 logMAR vs. 0.93 ± 0.19 logMAR, *p* = 0.05; Control, 1.15 ± 0.3 logMAR vs. 1.04 ± 0.22 logMAR, *p* = 0.08). However, the functional improvement was significant when pre-surgical visual acuity was compared to the last visual acuity in each group (Biofeedback: 0.35 ± 0.2 logMAR, *p* < 0.001; Control, 0.61 ± 0.35 logMAR, *p* = 0.001). The Biofeedback group had better baseline functional parameters including BCVA, retinal sensitivity, and fixation stability than the Control, though there was no statistical difference between the groups for all parameters. In both groups, BCVA significantly improved at all follow-up visits [(Biofeedback group: 3, 6, and 12 months, *p* = 0.002) (Control group: 3 months, *p* = 0.02; 6 and 12 months, *p* < 0.01)] compared to baseline. In the Biofeedback group, CRS significantly improved at the 6- (*p* = 0.03) and 12-month follow-ups (*p* = 0.01) from baseline, while a significant improvement for RS (*p* = 0.001) and MD (*p* = 0.005) was observed only at the last follow-up. In the Control group, only CRS (*p* = 0.01) significantly improved at the last follow-up (Figure 1).

Some patients in both the Biofeedback and Control groups showed a decrease in RS and CRS at the last follow-up. Specifically, 4 out of 12 patients in the Biofeedback group experienced a drop of sensitivity ranging from 0.5 dB to 1.8 dB, while 5 out of 11 patients in the Control group experienced a drop ranging from 0.3 dB to 5.8 dB (Table S1). 

**Figure 2:**
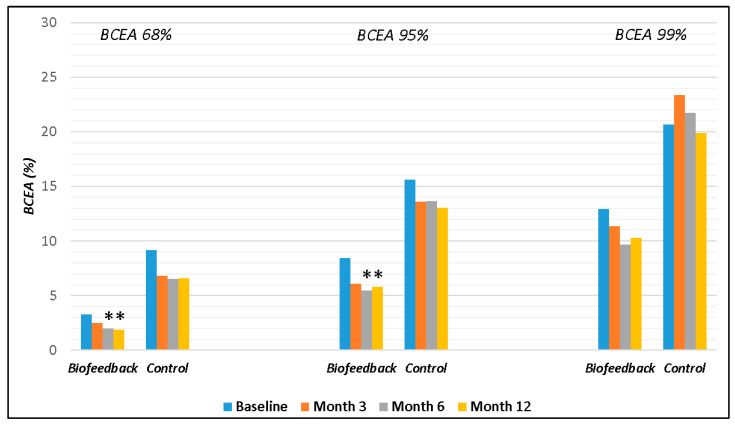
Changes in BCEA 68%, 95%, and 99% in Biofeedback and Control group. In the Biofeedback group, BCEA 68% and 95% significantly decreased at months 6 and 12. (BCEA, bivariate contour ellipse area); * *p* < 0.05 within each group. Each bar represents the mean value.

A significant decrease in the value of BCEA (by 68% and 95%) was observed in the Biofeedback group at the 6- and 12-month follow-ups. On the other hand, in the Control group, although there was a slight decrease in BCEA 68% and 95% at all follow-ups, it was not significant. However, an increase in BCEA 99% was observed at 3 and 6 months (Figure 2).

The comparison of functional parameters between the two groups at each follow-up revealed that RS, CRS, and BCEA 68%, 95%, and 99% were significantly better in the Biofeedback group than the Control group at months 3, 6, and 12. BCVA was significantly better in the trained group than the Control only at the 3-month follow-up. Lastly, MD was significantly better in the Biofeedback group at months 3 and 12 (Table 2).

The postoperative complications included mild ocular hypertension (IOP ≤ 25 mmHg) in five patients (Biofeedback group, n = 2; Control group, n = 3).

## 4. Discussion

This study examined the impact of a biofeedback training method that used visual and auditory stimuli on functional outcomes, such as visual acuity, retinal sensitivity, and a quantitative measure of fixation behavior (known as BCEA), in patients with closed hMMH after undergoing an inverted ILM-flap procedure. The results were compared to a control group that had similar anatomical outcomes and were monitored through scheduled visits. The biofeedback training gave significantly better functional outcomes, including retinal sensitivity within the central 4 and 12 degrees and fixation stability over a follow-up of 12 months from successful macular surgery. In line with previous studies [4,5,6,7,39], the mean visual acuity significantly improved after the inverted ILM-flap technique, as observed in both groups. The mean visual acuity was significantly better in the trained group only at month 3, and the final mean visual gain was not so different between the groups (Biofeedback, 0.57 logMAR; Control, 0.43 logMAR). The cataract extraction performed in all phakic eyes and the study population’s selection criteria, including the absence of retinal detachment and schisis with macular hole and an axial length ≥ 30 mm in only three cases, may justify the good visual acuity recovery as previously reported [40,41,42]. However, the visual acuity recovery did not reveal all functional changes related to the high myopic condition and surgical maneuvers. The high myopic condition [43,44,45], the loss of macular integrity due to the hole [18], and the morphologic changes after surgery [33,46,47] negatively influence retinal sensitivity and fixation behavior, which play an essential role in visual performance and could predict visual acuity recovery [33]. At baseline, microperimetry recorded a lower CRS than RS in both groups, revealing a deep central scotoma, which corresponds to the neurosensory defect of the macular hole, surrounded by a relative scotoma around the hole [46,47]. During the 12-month study, both groups showed improvement in the average values of retinal sensitivity parameters (RS, CRS, and MD). However, the Biofeedback group demonstrated significant improvement in RS and MD after the 12-month period, and in CRS after 6 and 12 months. On the other hand, the Control group showed significant improvement only in CRS at the last follow-up. Additionally, the trained group showed significantly better sensitivity parameters than the Control group throughout all follow-ups. Upon further analysis, we noticed a decrease in CRS and RS (Biofeedback: 33%, Control: 45%) during the last follow-up for some patients in both groups, regardless of their visual acuity improvement. However, the decrease was less significant in the trained group. There may be a correlation between improved sensitivity and cataract extraction [48], but the gliosis process caused by the inverted ILM-flap could negatively impact central sensitivity [33,46,47,49]. Additionally, ILM peeling may result in paracentral scotomata and reduced sensitivity within the central 12° due to temporary swelling in the arcuate nerve fiber layer [50]. The group that received biofeedback showed a significant improvement in sensitivity compared to the Control group. This suggests that combining biofeedback with an inverted ILM-flap technique may help preserve and enhance retinal sensitivity. Similar results were observed in prior studies when microperimetric training was performed after macular surgery [20,28,29]. Fixation stability is another functional parameter to be considered, probably more than the fixation location because the fixation site could already be naturally relocated out of the fovea [8,9,10,51,52]. BCEA significantly improved with a reduction in the dimension of the innermost internal areas of the fixation points only in the Biofeedback group at 6 and 12 months, and the trained group achieved better fixation than the Control at all follow-ups. Patients typically adapt within a few months to using an eccentric locus for fixation [8,53], and also they can develop a PRL in a not helpful location for left-to-right reading [54]. So, we encouraged the patients to fixate on their PRL in a convenient location and within an area of adequate retinal sensitivity. Otherwise, we selected a TRL, which was preferentially located within an area of good retinal sensitivity, not too far from the central fovea and in a convenient location. Extensive training [55] and neuro-psychology rehabilitative procedures, such as microperimetric biofeedback, can help patients effectively use a new locus of fixation [8]. This includes training patients to perform eccentric viewing, which can increase retinal sensitivity and stabilize fixation, ultimately improving visual performance [8]. It is not clear how the modification of the locus of fixation and the improvement in visual perception are achieved. One possible explanation is that training redirects the oculomotor system to a location with better visual sensitivity [56]. When the retina suffers from local damage, the affected area cannot be stimulated, but cortical neurons that are usually driven by stimuli in this area remain active and respond selectively to stimulation from other parts of the retina [57,58]. As previously observed, combining structured and acoustic stimuli increases the function of PRL [21,22,28]. Acoustic feedback can help strengthen the PRL by increasing attentional modulation, while the structural light stimulus targets visual receptive fields that are highly sensitive to medium spatial frequencies [15]. Microperimetric biofeedback, which provides auditory feedback, may aid in maintaining target stimulation of the retina, thereby strengthening the patient’s cortical plasticity and facilitating neural signals within the retina and between the retina and brain [25].

Our biofeedback protocol consisted of 12 training sessions, each lasting 10 min, twice a week. Until now, research that aims to determine the most optimal protocol is still being carried out. Several studies reported differences in intensity, frequency, and duration of training with different but satisfactory rehabilitation outcomes [20,27,29,59,60]. Some authors reported that visual performance could be maintained by performing follow-up training sessions [27,29]. However, the duration effect of biofeedback on visual function remained vague and more extensive studies with longer follow-up are required. Eccentric viewing training has been found to offer greater benefit in terms of improved near-vision tasks and vision-related quality of life [61,62]. Thus, due to its non-invasive nature and quick execution, it has the potential to become a standard post-surgery procedure. There were no studies that included an economic evaluation of eccentric viewing training including biofeedback training strategy. However, it is possible to theorize that there could be positive socioeconomic effects for patients who experience an improvement in their visual function.

One of the strengths of this study is that it compares a well-defined population of patients who have undergone surgical resolution of hMMH. It should be mentioned that the Biofeedback group, which consisted of patients who had undergone surgery more recently, showed better pre-surgical and baseline parameters compared to the Control group. However, this difference did not reach statistical significance. The improvement in the Biofeedback group could be due to the surgeon’s enhanced surgical skills. The study also followed a standardized protocol of biofeedback rehabilitation and had a long-term follow-up. However, the study’s limitations include the retrospective nature of the study, a small sample size, the absence of analysis of the restoration of the ellipsoid zone and external limiting membrane, and their relationship with functional changes. In this regard, recent studies have shown that the status of the outer retinal layers is connected to visual function, such as visual acuity [63], retinal sensitivity, and fixation behavior [49]. However, it is important to note that the analysis of structural parameters was based solely on the horizontal scans of OCT images, while scans from all directions should be considered. Additionally, the measurement of morphologic parameters such as the diameter of the ellipsoid zone and external limiting membrane defect, as well as their thickness and reflectivity before and after surgery, must be standardized, especially when using image analysis software outside of the OCT device [63]. Lastly, our study did not analyze any changes in vision-related quality of life after rehabilitation training, and there may have been some measurement error or intrinsic variability in the microperimetric test [22].

## 5. Conclusions

The visual acuity and retinal sensitivity improvement also confirmed the effectiveness of the inverted ILM-flap technique on functional recovery. After a successful inverted ILM-flap surgery for hMMH, using microperimetric biofeedback can enhance retinal sensitivity and improve fixation stabilization beyond the standard care strategy. Further research is required to study the effect of this rehabilitation training on daily activities.

## Figures and Tables

**Table 1:** Pre-operative characteristics of patients.

Parameters *	Biofeedback Group (n = 12)	Control Group (n = 11)	*p* ^§^
Gender (M) (%)	10 (83.33)	4 (36.36)	0.04 ^^^
Age (yrs)	63.25 ± 8.79	68.00 ± 7.22	0.17
AL (mm)	26.72 ± 1.09	27.89 ± 2.40	0.04
Hole size (µm)	347.00 ± 115.84	404.73 ± 186.49	0.45
Lens status (%)			0.64 ^^^
Phakic	10 (83.33)	8 (72.73)	
Pseudophakic	2 (16.67)	3 (27.27)	
RE in phakic eyes (D)	−8.35 ± 2.90	−11.93 ± 7.80	0.35
BCVA pre-surgical (logMAR)	1.03 ± 0.11	1.15 ± 0.30	0.21

* As mean and standard deviation (M ± SD) for continuous variables, and percentage (%) for categorical variables. Abbreviations: AL, axial length; RE, refractive error (spherical equivalent); BCVA, best-corrected visual acuity; § Wilcoxon rank-sum (Mann–Whitney) test; ^ Chi square test.

**Table 2:** Comparison of clinical parameters between the groups over follow-up.

Parameters *	Control Group				Biofeedback Group				*p* ^	*p* ^ѱ^	*p* ⸹	*p* ^†^
	Baseline	3 Months	6 Months	12 Months	Baseline	3 Months	6 Months	12 Months				
BCVA (logMAR)	1.04 ± 0.22	0.83 ± 0.34	0.63 ± 0.31	0.61 ± 0.35	0.92 ± 0.19	0.51 ± 0.24	0.40 ± 0.23	0.35 ± 0.22	0.20	0.01	0.22	0.09
BCEA 68%	9.20 ± 8.78	6.79 ± 6.68	6.54 ± 7.32	6.61 ± 7.44	3.27 ± 2.61	2.51 ± 2.14	1.98 ± 2.07	1.85 ± 2.15	0.05	0.008	0.002	0.0003
BCEA 95%	15.60 ± 13.42	13.58 ± 9.67	13.66 ± 10.89	13.02 ± 9.81	8.44 ± 5.86	6.08 ± 5.58	5.47 ± 5.59	5.80 ± 5.79	0.15	0.007	0.01	0.006
BCEA 99%	20.68 ± 12.24	23.34 ± 11.28	21.73 ± 11.89	19.86 ± 10.2	12.91 ± 9.35	11.37 ± 10.17	9.67 ± 9.56	10.26 ± 9.92	0.10	0.003	0.001	0.004
RS (dB)	9.54 ± 5.10	9.77 ± 4.59	10.61 ± 4.82	10.16 ± 4.23	13.32 ± 4.02	14.48 ± 2.60	14.80 ± 2.71	15.80 ± 2.59	0.11	0.02	0.04	0.0007
MD (dB)	−9.50 ± 4.73	−9.15 ± 4.17	−8.03 ± 4.64	−8.76 ± 4.34	−6.02 ± 4.05	−5.05 ± 2.64	−4.73 ± 2.81	−3.67 ± 2.72	0.09	0.01	0.09	0.003
CRS (dB)	6.56 ± 4.06	7.12 ± 4.35	7.83 ± 4.52	8.73 ± 4.45	10.07 ± 4.88	11.45 ± 4.81	12.26 ± 3.89	13.55 ± 4.20	0.11	0.02	0.02	0.008

* As mean and standard deviation (M ± SD). Abbreviation: BCVA, best-corrected visual acuity; BCEA, bivariate contour ellipse area which contains 68% or 95% or 99% of fixation points; RS, retinal sensitivity; MD, mean deviation; CRS, central retinal sensitivity. ^ Wilcoxon rank-sum test (Mann–Whitney) compared to baseline; ^ѱ^ Wilcoxon rank-sum test (Mann–Whitney) compared to 3 months; ⸹ Wilcoxon rank-sum test (Mann–Whitney) compared to 6 months; ^†^ Wilcoxon rank-sum test (Mann–Whitney) compared to 12 months.

## Data Availability

The data that support the findings of the present study are available from the corresponding author (A.N.) upon reasonable request.

## Supplementary Materials

The following supporting information can be downloaded at: https://www.mdpi.com/article/10.3390/jcm12165188/s1, Table S1: Retinal sensitivity parameters (RS, Retinal Sensitivity; MD, Mean Deviation; CRS, Central Retinal Sensitivity) for each patient in both groups over follow-up.
