# Supplementary material for: Biofeedback Training after Successful Inverted Internal Limiting Membrane (ILM)-Flap Technique for High Myopic Macular Hole
Source: J Clin Med. 2023 Aug 9;12(16):5188. doi: 10.3390/jcm12165188 (PMC10455115; doi:10.3390/jcm12165188)
Supplement: Supplementary file 1 [file jcm-12-05188-s001.zip › jcm-2495460-supplementary.pdf]

Table S1. Retinal sensitivity parameters (RS, Retinal Sensitivity; MD, Mean Deviation; CRS, Central Retinal Sensitivity) for each patient in both groups over follow-up

Control Group

RS (Baseline)	RS (3 m)	RS (6 m)	RS (9 m)
13.4	10	11.6	11.1
7	8.2	7.4	7.9
2.6	3.4	3.3	3.1
15.8	10.1	11.8	10
16.3	17.5	18.4	14.1
10.2	7.3	6.9	7.1
10.9	14.4	14.6	15.2
14.1	13.9	14.2	14.2
8.2	10.6	13.9	12.9
4.2	10.2	11.2	12.6
2.3	1.9	3.4	3.6

MD (baseline)	MD (3m)	MD (6m)	MD (9m)
-5.9	-9.9	-8.2	-8.7
-12.6	-11.7	-12.4	-12.7
-14.8	-13.5	-13.6	-13.8
-3.2	-7.5	-6.1	-9.9
-3.5	-2.3	-1.4	-5.6
-8.9	-11.8	-12.3	-11.9
-7.8	-4.5	-4.9	-4.3
-5.4	-4.9	-4.7	-4.7
-11.5	-9.2	-6	-7
-14.3	-9	-3.4	-2.3
-16.6	-16.3	-15.3	-15.5

CRS (Baseline)	CRS (3m)	CRS(6m)	CRS (9m)
9.1	9	10	10.9
7.3	7.2	6.9	7.89
0.7	1	1.2	1.1
8.3	6.7	7.2	13.2
12.4	16.8	16.5	13.8
4.5	4.6	3.9	4.2
10.5	10.4	11.4	13.5
10.3	7.9	8.1	8.2
6.2	8.2	12.4	12.1
1.9	4.9	5.7	8.2
1	1.6	2.8	2.9

Table S1. Retinal sensitivity parameters (RS, Retinal Sensitivity; MD, Mean Deviation; CRS, Central Retinal Sensitivity) for each patient in both groups over follow-up

Biofeedback Group

RS (Baseline)	RS (3m)	RS (6m)	RS (9m)
15.4	16.98	17.89	18.2
8.8	16.3	15.2	15.9
19.2	18.4	19.6	19.8
12.8	12.8	16.5	17
14.9	13.3	13.5	13.1
12.7	16.7	17	17.4
16	12.7	13.5	14.6
15.8	17.5	16	18.1
15.7	13.7	12.8	14.9
14.4	12.8	13.6	13.9
4.1	9.8	10.2	10.4
10.1	12.8	11.8	16.3

MD (Baseline)	MD (3m)	MD (6m)	MD (9m)
-2.5	-1.9	-0.8	-0.7
-11.1	-4.2	-4.6	-3.9
-0.7	-1.1	-0.2	0
-6.9	-6.8	-3.3	-2.8
-3	-4.8	-3.8	-4.2
-6.9	-2.7	-2.8	-2
-3.8	-6.8	-5.9	-4.5
-4	-2.3	-3.7	-0.8
-4.1	-6.3	-7.1	-4.8
-5.1	-7	-8.4	-8.6
-14.9	-9.8	-8.4	-8.2
-9.3	-6.9	-7.8	-3.5

CRS (Baseline)	CRS (3m)	CRS (6m)	CRS (9m)
8.2	9.1	11.2	13.9
6.4	15.6	12.9	16.6
19.5	17	19.5	20
12.1	12.8	15.8	14.6
12.4	12.2	11.9	11
8.2	11.7	11.8	12.6
13.4	12.4	12.6	12.5
11.2	18.3	17	18.5
15	13.3	11.7	15.8
7.4	8.3	8.5	8.2
0.9	5.1	4.9	4.9
6.2	1.6	9.3	14
